# Serodiagnosis of equine infectious anemia by indirect ELISA based on a novel synthetic peptide derived from gp45 glycoprotein

**DOI:** 10.1007/s11259-025-10707-x

**Published:** 2025-04-22

**Authors:** Aníbal Domínguez-Odio, Laura Iliana Coroas González, Dayamí Martín Alfonso, Francisco Guevara-Hernández, Manuel Alejandro La O Arias, Mayelin Paneque Zayas, Miguel Ángel Bedoya Ríos

**Affiliations:** 1 Dirección de Ciencia e Innovación. Grupo Empresarial LABIOFAM, Avenida Independencia km 16 ½, La Habana, Cuba; 2 Centro de Investigaciones Científicas de la Defensa Civil, Carretera de Tapaste y Autopista Nacional Km 23 ½, San José de las Lajas, Cuba; 3 Facultad de Ciencias Agronómicas, Universidad Autónoma de Chiapas, Carretera Ocozocuautla-Villaflores Km. 84.5, Villaflores, México; 4 Animal Science Research Group, Universidad Cooperativa de Colombia, sede Bucaramanga. Carrera 33 N°. 30ª-05 (4.162,49 km, Bucaramanga, 68000 Colombia

**Keywords:** Equine infectious anemia, ELISA test, Synthetic peptide, Serological diagnosis

## Abstract

The transmembrane glycoprotein gp45 (implicated in virus-equine B lymphocyte binding and in subsequent infection) is an important protein used as an antigen in the serological diagnosis of equine infectious anemia, but synthetic peptides derived from its structure are not. Consequently, the present study aimed to determine the diagnostic performance of the Bio-AIELAB immunoassay (novel synthetic peptide P05/gp45) in comparison with a similar commercial assay (ELISA EIA, VMRD Inc, USA) and with the gold standard (AGID, LABIOFAM, Cuba). A total of 365 samples of sera from naturally infected Cuban horses (positive: 179, and negative: 186) were investigated by Bio-AIELAB and AGID, of which 299 (positive: 147, and negative: 152) were analysed by ELISA EIA. The performance indicators used in all cases were: relative diagnostic sensitivity and specificity, positive and negative predictive values, as well as concordance according to the Kappa index. The Bio-AIELAB system obtained the best performance in sensitivity (99.44%, 178/179), specificity (95.16%, 177/186) and concordance (Kappa index: 0.94) with respect to ELISA EIA test. Based on the above elements, Bio-AIELAB could be a promising candidate for use in equine infectious anemia surveillance programs in combination with the AGID test.

**Supplementary Information:**

The online version contains supplementary material available at 10.1007/s11259-025-10707-x.

## Introduction

The horse (*Equus caballus*, Lin 1758), perhaps like no other domesticated species, holds high military, cultural, sports, recreational, and economic value (transportation, agricultural and livestock work, meat, milk, hair, and leather) (Merkies and Franzin 2021). Consequently, it is a health necessity to diagnose and appropriately treat the infectious diseases that affect them, particularly equine infectious anemia (EIA) (Jara et al. 2020; World Organisation for Animal Health (WOAH), 2019).

The latter stands out among all, as there are no available treatments or preventive vaccines, leading to significant economic losses due to the elimination of positive animals and international restrictions, including exhibitions, horse competitions, and sperm commercialization (Cook et al. 2013). These reasons compel the development of new diagnostic tools as a cost-effective way to reduce the virus’s transmissibility to healthy populations (Machado et al. 2021).

In this context, enzyme-linked immunosorbent assays (ELISA) emerge as an attractive solution in cases where a massive, rapid, automated (Hu et al. 2023) and more sensitive screening is required (Scicluna et al. 2013). These advantages favor their use in combination with the agar gel immunodiffusion test (AGID), the latter intended to confirm positive ELISA results (WOAH, 2019). This diagnostic approach led to the use of antigens derived from three structural viral proteins: the capsid protein p26, the transmembrane glycoprotein gp45 and the surface glycoprotein gp90 (Issel et al. 2013). On that basis, several ELISAs using recombinant p26 (Nardini et al. 2017), recombinat gp45 (Du et al. 2018), recombinat gp90 (Reis et al. 2012), but only a few use synthetic peptides (Russi et al. 2023) are frequently reported.

The international scientific trend towards the use of specific molecules as antigens to detect EIA infections allows inferring the relevance of research linked to synthetic peptides. The identification of new and improved immunodominant epitopes of the virus will allow first, to expand the possibilities of combinations (single or mixtures) to improve the serological diagnosis of the disease and second, to increase the commercial catalog of products from small, stable, easy to synthesize and manipulate chemical structures (Heyduk et al. 2018). For these reasons, the present study aimed to determine the diagnostic performance of ELISA Bio-AIELAB based on the novel synthetic P05/gp45 peptide in comparison with the similar commercial assay and the gold standard (confirmatory) in sera from horses naturally infected with EIA.

## Materials and methods

### Synthetic peptide

To predict the antigenicity of the amino acid sequence of the *env* gene, the DNAstar bioinformatics tool (Madison, Wisconsin, USA) was used. Analysis of sequencing data identified two candidate peptide sequences (P05 and P15) derived from glycoproteins gp45 and gp90, respectively. However, only P05 showed the highest capacity to detect EIA-specific antibodies (unpublished results), and its sequence had not been previously described in the literature (Soutullo et al. 2007; Santos et al. 2012; Naves et al. 2019; Ostuni et al. 2023). The gp45 originate from a wild pathogenic strain circulating in Cuba, whose partial sequence is available in GeneBank with accession number HQ853234.1 (Díaz-Miranda et al. 2012).

The synthetic structure (folded form composed of a sequence of 26 aminoacids) was obtained at the Center for Genetic Engineering and Biotechnology (Cuba) through solid-phase chemical synthesis using the Boc strategy in porous polypropylene bags. Each 30 × 40 mm bag contained 60 mg of polymeric polystyrene resin with 1% divinylbenzene as support. After sealing and coding, the specific amino acids were gradually incorporated in repeated coupling cycles, after deprotection of the α-amino group. At the end of the assembly, the side chains were deprotected and the peptide was separated from the support according to Amblard (2006). Subsequently, they were purified by high-performance liquid chromatography in reverse-phase (RP-HPLC, Pharmacia, LKB) with RP18 column (A/B gradient system from 0 to 60%, A: TFA 0,1% (v/v) and acetonitrile 2% (v/v) in H2O; B: TFA 0,05% (v/v) in acetonitrile), obtaining values higher than 85.0% (Hernández et al. 2001).

### Collection of clinical samples

**Fig. 1:**
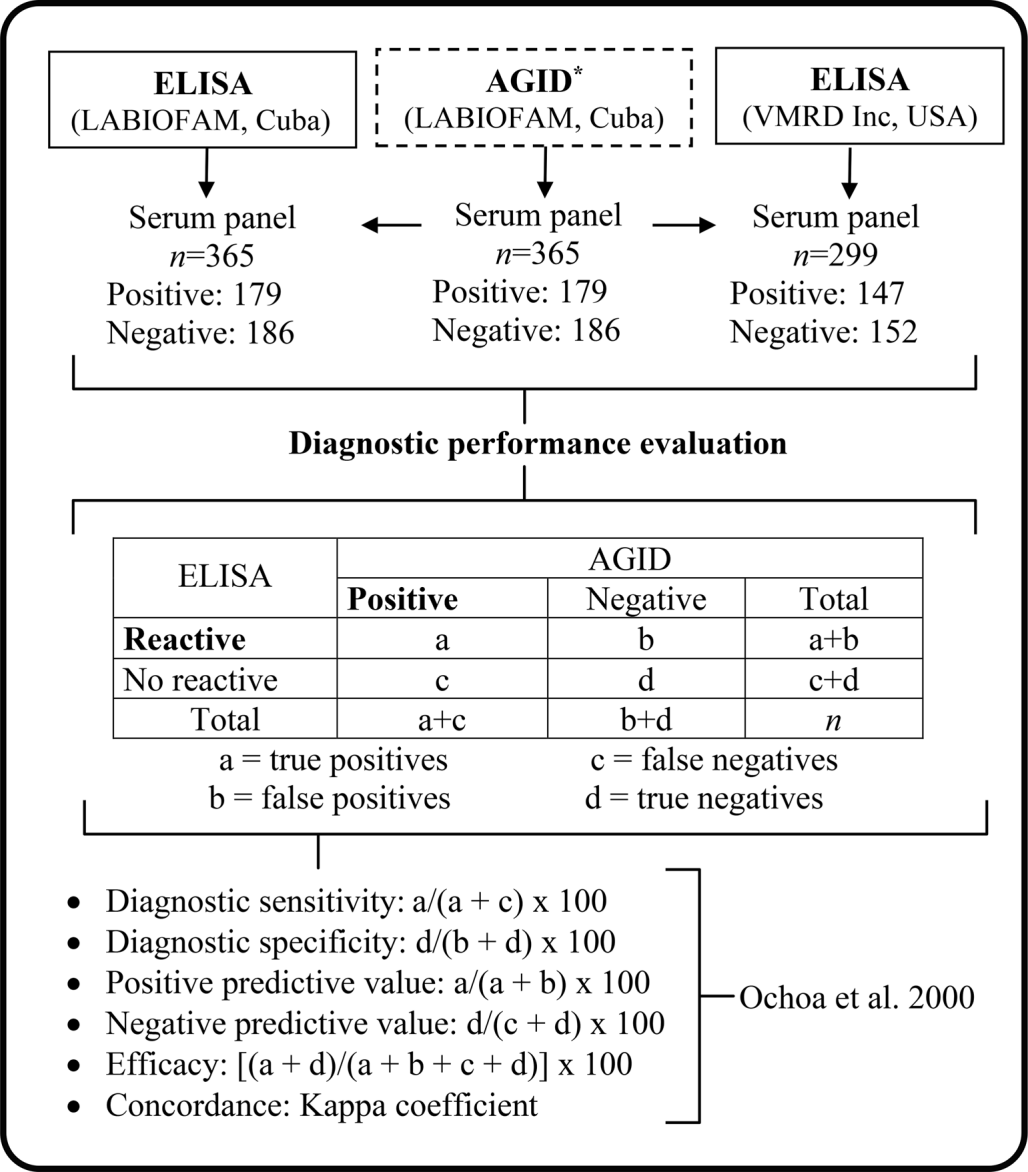
General aspects of the study. Note: *Reference test for the WOAH

The serological testing in general was carried out with samples of 664 equine serum (positive: 326, and negative: 338) donated by the Central Unit of Agricultural Health Laboratories of Cuba and previously classified by the AGID technique. In particular, ELISA Bio-AIELAB (LABIOFAM, Cuba) evaluated 365 serum, while ELISA EIA (VMRD Inc, USA) evaluated 299 sera as shown in Fig. 1. All samples collected from naturally infected animals, showing heterogeneous clinical states. The collected sera were stored at −40°C until further processing. No experimental infections occurred in any case.

#### ELISA Bio-AIELAB (LABIOFAM, Cuba)

MaxiSorp polystyrene microplates (Thermo Fisher Scientific, USA) were sensitized with the P05 peptide (patent pending) using 100 µL/well dissolved at 1 µg/mL in phosphate buffer saline (PBS) 1×, and incubated for 20 h at 4°C. Subsequently, each microplate was washed with phosphate buffered saline - Tween 20 (PBS-T) 1×, and blocked (skim milk 3%) for 1 h at 37°C. The plates coated and labelled with P05 were covered with adhesive tape and stored at 2–8°C until their use.

Once ready, two wells were prepared with standard positive serum, standard negative serum and each serum to be tested. In all cases, 5 µL of serum and 95 µL of diluent (PBS) were added per well. The microplates were gently shaken to facilitate homogenization and incubated for 1 h at 37°C ± 2°C. Then, the wells were then washed six times with PBS-T 1× using an ELISA microwasher (SUMA, Cuba) adding 300 µl/well. Subsequently, 100 µL of the conjugate (sheep anti-IgG equine conjugated with horseradish peroxidase, CICDC, Cuba) diluted 1:10,000 in PBS was added. Each microplate was then incubated for 1 h at 37°C ± 2°C in a humid chamber, washed four times using a washing solution. After this, 100 µL of tetramethyl benzidine substrate (Sigma-Aldrich, USA) was added to each well, and the microplates were incubated in the dark at room temperature for 15 min. The reaction was stopped using 100 µL of 2 M H_2_SO_4_. The microplates were dried and the absorbance was read at 492 nm in a PR-521 ELISA reader (SUMA, Cuba).

The interpretation of the results was performed taking into account the cut-off point, previously calculated by analyzing 365 AGID-confirmed serum (179 positive and 186 negative). The optical density (OD) readings obtained at 492 nm were adjusted by ROC (receiver operator characteristic) curve analysis (Cerda and Cifuentes 2012) using the bioinformatics tool WinEpiscope 2.0 (Thrusfield et al. 2001). For an area under the curve of 97.57% (lower limit: 96.67% and upper limit: 98.47%), OD 0.300 was identified as the cut-off point and on this basis OD values ≥ 0.30 were estimated to be reactive, those ≤ 0.25 nonreactive and the range 0.25–035 as the gray zone.

#### ELISA EIA (VMRD Inc, USA)

Modified sandwich-type system designed for the detection of specific antibodies against equine infectious anemia virus in serum, designed for 96 determinations. The strength of this kit lies in the presence of the recombinant p26 antigen of the EIA virus (EIAv), capable of binding to anti-EIA antibodies present in infected animals. All components were used according to the manufacturer’s instructions.

### Gold standard

#### AGID-AIE (LABIOFAM, Cuba)

Petri dishes with a diameter of 90 mm and 15 mL of 1% agar were utilized. Once solidified, the agar was perforated with a mold that created a central well and six peripheral wells. The dimensions were 5.3 mm in diameter and 2.4 mm distance between wells. The p26 antigen protein (24 µL) was placed in the central well, and positive control sera interspersed with diagnostic target serum samples (24 µL) were placed in the peripheral wells. AGID test results were interpreted as positive through visual reading of the curvature of the precipitation line or as negative due to the absence of the line, after 48 and 72 h of incubation at a temperature between 20 and 25°C. AGID tests were considered valid only if the negative and positive controls included in each test plate yielded expected results. Samples were reanalyzed when the interpretation of the results was ambiguous (Bannai et al. 2023).

### Analysis of data

After serological evaluation at each stage, the following quantitative parameters were determined (Fig. 1): sensitivity, specificity, positive predictive value, negative predictive value, precision and Kappa coefficient according to Ochoa et al. (2000). The strength of agreement with kappa values was interpreted as poor, < 0.20; fair, 0.21 to 0.40; moderate, 0.41 to 0.60; good, 0.61 to 0.80; or very good, 0.81–1.00. The processing of the data corresponding to sensitivity, specificity, positive predictive value, negative predictive value and efficacy obtained in the two ELISAs was used using Fisher’s exact test for comparison of proportions and a statistically significant value was considered when *p* < 0.05. The statistical analysis was performed with the use of SPSS statistics software (version 23.0).

## Results

Table 1 shows the comparative results between the two ELISAs and the AGID assay, revealing that Bio-AIELAB and ELISA EIA show different performances. The first system for example, achieved an excellent coincidence in the detection of antibodies in the positive horse sera (178/179) with respect to the reference test, while the same did not occur with the EIA ELISA system, which failed to correctly identify 8 samples declared as positive by AGID (139/147). In spite of the differences identified between the two ELISAs, it was demonstrated that in general terms they have a high efficacy, which in our case was higher than 96%. This aspect is satisfactory for their inclusion in the initial phases of the diagnostic algorithm.

**Table 1:** Diagnostic performance parameters of the developing and commercial ELISAs with respect to AGID

Bio-AIELAB	AGID EIA, Cuba				Diagnostic performance indicators	Significance (*P* value)
	**Positive Negative Total**					
Reactive	178	9		187	Diagnostic sensitivity: 99.4%	0.013*
					Diagnostic specificity: 95.1%	ns
No reactive	1	177		178	Positive predictive value: 83.2%	0.017*
					Negative predictive value: 99.4%	0.014*
Total	179	186		**365**	Efficacy: 97.2%	ns
					Kappa coefficient: 0.94 (very good)	
ELISA EIA						
Reactive	139		3	142	Diagnostic sensitivity: 94.5%	0.013*
					Diagnostic specificity: 98.0%	ns
No reactive	8		149	157	Positive predictive value: 97.8%	0.017*
					Negative predictive value: 94.9%	0.014*
Total	147		152	**299**	Efficacy: 96.3%	ns
					Kappa coefficient: 0.92 (very good)	

*Significance when applying Fisher's exact comparison test, ns: no significant differences exist

The statistical analysis of each indicator revealed that Bio-AIELAB, in relation to EIA ELISA, has a significantly higher probability of identifying with certainty infected animals (99.4%) and of declaring the healthy animal as non-reactive (99.4%), according to Fisher’s exact test for comparison of proportions. It was also found that the EIA ELISA system has a significantly higher capacity to declare the animal truly infected as reactive (97.8%) compared to Bio-AIELAB. The kappa index values of 0.94 and 0.92 showed in both cases an excellent level of agreement between ELISAs and AGID. The value of the kappa statistic, close to 1, indicates a very good concordance between the diagnostic techniques beyond chance.

## Discussion

In general terms, the satisfactory performance visualised in Bio-AIELAB demonstrates the usefulness of using the synthetic peptide and the transmembrane glycoprotein gp45 as antigen to identify EIA-specific antibodies (Du et al. 2018; Fontes et al. 2018; Naves et al. 2019; Aguilar-Montes de Oca 2022). The high levels of sensitivity shown in this case can be interpreted as an ability to identify antibody response, which is essential to avoid the persistence of EIA (Scicluna et al. 2013).It should be noted that the high sensitivity and diagnostic specificity values obtained by Bio-AIELAB in the present study are equivalent or even higher than those reported by their international counterparts (Reis et al. 2012; Alvarez et al. 2015; Nardini et al. 2017; Scicluna et al. 2018; Fontes et al. 2018; Du et al. 2018; Naves et al. 2019; Russi et al. 2023).

Table 2 shows the ability of the Bio-AIELAB to obtain an adequate diagnostic profile. The comparative analysis of the sensitivity-specificity indicators as a whole showed that in some cases it was superior to other indirect ELISAs stated by Naves (2019) in Brazil, while in others it showed similar figures to those expressed by Alvarez (2015) in Argentina. The P05 peptide performed better in the sensitivity indicator with respect to other synthetic and recombinant gp45 and gp90 antigens reported by Naves (2019); Du (2018) and Reis (2012). Favorable as well were the specificity values obtained in this study, which exceeded what was published for the recombinant p26 protein (Alvarez et al. 2015) and the synthetic peptide combinations derived from gp90 and gp45 reported by Russi (2023).

**Table 2:** Diagnostic performance of different ELISAs (commercial or under development) for equine infectious Anemia

Antigens	Sensitivity	Specificity	Serum samples	References
	%		Positive/Negative	
Synthetic peptide gp45^b^	98.6	95.6	*n* = 859 (143/716)	Naves et al. 2019
Recombinant gp45^b^	90.0	99.3	*n* = 546 (14/532)	Du et al. 2018
Recombinant p26^a^	100	100	*n* = 30 (22/8)	Nardini et al. 2017
Recombinant p26^a^	100	94.3	*n* = 302 (93/209)	Alvarez et al. 2015
Recombinant p26^b^	100	100	*n* = 569 (288/281)	Fontes et al. 2018
Chimeric and peptide^a^	100	99.3	*n* = 615 (70/545)	Scicluna et al. 2018
Recombinant gp 90^b^	96.1	96.4	*n* = 1160 (179/981)	Reis et al. 2012
Synthetic peptides gp90 and gp45^b^	99.5	90.3	*n* = 1121 (243/878)	Russi et al. 2023

^a^commercially available internationally

^b^system in developmental phase

The variety of molecules and combinations used in commercial or investigational ELISAs for the diagnosis of EIAv is associated with the multiple options generated by the proteins that constitute their structure (Cook et al. 2013). The selection of each contributes to the singularity of each analytical system and, at the same time, causes variability in the results (Table 2), demonstrating that this is a critical element for enzyme-linked immunosorbent assays. (Bueno et al. 2020; Pandey et al. 2021). In our case, the favourable performance of the P05 antigenic peptide, in relation to that used by Naves (2019) and the combination of two peptides designed by Russi (2023) allows inferring that the novel Cuban peptide exhibits elevated reactivity associated with its high homology with specific regions in antibodies against EIAv (patent pending).

At the industrial level, the use of the short peptide as a serological diagnostic antigen provides several additional advantages such as chemical stability, and relative ease by which they can be synthesized and manipulated (Heyduk et al. 2018). Its acquisition avoids technical problems that arise when attempting to purify liposoluble recombinant molecules and eliminates the need to cultivate the virus and inoculate it into susceptible animals, which are often laborious, time-consuming, costly, and ethically discouraged processes (Fontes et al. 2018).

The results of the present study demonstrated that the Bio-AIELAB indirect ELISA can be a useful alternative diagnostic tool and can be included in surveillance and control programs for equine infectious anemia in combination with the AGID test.

## Supplementary Information

Supplementary Material 1

Supplementary Material 2

## Data Availability

No datasets were generated or analysed during the current study.
