# Supplementary material for: Serodiagnosis of equine infectious anemia by indirect ELISA based on a novel synthetic peptide derived from gp45 glycoprotein
Source: Vet Res Commun. 2025 Apr 22;49(3):174. doi: 10.1007/s11259-025-10707-x (PMC12014826; doi:10.1007/s11259-025-10707-x)
Supplement: Supplementary file 1 — Supplementary Material 1 [file 11259_2025_10707_MOESM1_ESM.doc]

Table 1: Diagnostic performance parameters of the developing and commercial ELISAs with respect to AGID

| Bio-AIELAB | AGID EIA, Cuba | | | | | | | | Diagnostic performance Significance  indicators (P value) | | |
| --- | --- | --- | --- | --- | --- | --- | --- | --- | --- | --- | --- |
|  | **Positive Negative Total** | | | | | | | |  |  |  |
| Reactive | 178 | 9 | | | 187 | | | Diagnostic sensitivity: 99.4% 0.013* | | | |
|  |  |  |  |  |  |  |  | Diagnostic specificity: 95.1% ns | | | |
| No reactive | 1 | 177 | | | 178 | | | Positive predictive value: 83.2% 0.017* | | | |
|  |  |  |  |  |  |  |  | Negative predictive value: 99.4% 0.014* | | | |
| Total | 179 | 186 | | | **365** | | | Efficacy: 97.2% ns | | | |
|  |  |  |  |  |  |  |  | Kappa coefficient: 0.94 (very good) | | | |
| ELISA EIA |  | | |  | |  | | | |  | |
| Reactive | 139 | | 3 | | 142 | | Diagnostic sensitivity: 94.5% 0.013* | | | |  |
|  |  |  |  |  |  |  | Diagnostic specificity: 98.0% ns | | | |  |
| No reactive | 8 | | 149 | | 157 | | Positive predictive value: 97.8% 0.017* | | | |  |
|  |  |  |  |  |  |  | Negative predictive value: 94.9% 0.014* | | | |  |
| Total | 147 | | 152 | | **299** | | Efficacy: 96.3% ns | | | |  |
|  |  |  |  |  |  |  | Kappa coefficient: 0.92 (very good) | | | |  |

Note: *Significance when applying Fisher's exact comparison test, ns: no significant differences exist

Table 2: Diagnostic performance of different ELISAs (commercial or under development) for Equine Infectious Anemia

| Antigens | Sensitivity | Specificity | Serum samples | References |
| --- | --- | --- | --- | --- |
|  | % | | Positive/Negative |  |
| Synthetic peptide gp45^**^ | 98.6 | 95.6 | n=859 (143/716) | Naves et al. 2019 |
| Recombinant gp45^**^ | 90.0 | 99.3 | n=546 (14/532) | Du et al. 2018 |
| Recombinant p26^*^ | 100 | 100 | n=30 (22/8) | Nardini et al. 2017 |
| Recombinant p26^*^ | 100 | 94.3 | n=302 (93/209) | Alvarez et al. 2015 |
| Recombinant p26^**^ | 100 | 100 | n=569 (288/ 281) | Fontes et al. 2018 |
| Chimeric and peptide^*^ | 100 | 99.3 | n=615 (70/545) | Scicluna et al. 2018 |
| Recombinant gp 90 ^**^ | 96.1 | 96.4 | n=1160 (179/981) | Reis et al. 2012 |
| Synthetic peptides gp90 and gp45^**^ | 99.5 | 90.3 | n=1121 (243/878) | Russi et al. 2023 |

Note: *commercially available internationally, **system in developmental phase
