# Supplementary material for: Serodiagnosis of equine infectious anemia by indirect ELISA based on a novel synthetic peptide derived from gp45 glycoprotein
Source: Vet Res Commun. 2025 Apr 22;49(3):174. doi: 10.1007/s11259-025-10707-x (PMC12014826; doi:10.1007/s11259-025-10707-x)
Supplement: Supplementary file 2 — Supplementary Material 2 [file 11259_2025_10707_MOESM2_ESM.doc]

Serum panel

*n*=299

Positive: 147

Negative: 152

**Diagnostic performance evaluation**

**ELISA**

(LABIOFAM, Cuba)

**AGID^*^**

(LABIOFAM, Cuba)

**ELISA**

(VMRD Inc, USA)

Serum panel

*n*=365

Positive: 179

Negative: 186

Serum panel

*n*=365

Positive: 179

Negative: 186

Diagnostic sensitivity: a/(a + c) x 100

Diagnostic specificity: d/(b + d) x 100

Positive predictive value: a/(a + b) x 100

Negative predictive value: d/(c + d) x 100

Efficacy: [(a + d)/(a + b + c + d)] x 100

Concordance: Kappa coefficient

Ochoa et al. 2000

ELISAAGID**Positive**NegativeTotal**Reactive**aba+bNo reactivecdc+dTotala+cb+d*n* a = true positives c = false negatives

b = false positives d = true negatives

Figure 1. General aspects of the study

Note: *Reference test for the WOAH
